# Association between altered placental human chorionic gonadotrophin (hCG) production and the occurrence of cryptorchidism: a retrospective study

**DOI:** 10.1186/1471-2431-14-191

**Published:** 2014-07-26

**Authors:** Carole Chedane, Hugues Puissant, Dominique Weil, Stéphanie Rouleau, Régis Coutant

**Affiliations:** 1 Department of Pediatric Endocrinology, University Hospital, 4 rue Larrey, 49033 Angers CEDEX 01, France; 2 Department of Genetics, University Hospital, Angers, France; 3 Department of Pediatric Surgery, University Hospital, Angers, France

**Keywords:** Cryptorchidism, Males, hCG

## Abstract

**Background:**

An increase in cryptorchidism has been reported in many countries. One mechanism could be low fetal testosterone production possibly secondary to altered placental human chorionic gonadotrophin (hCG) release. Our Objective was to compare hCG values from maternal blood between boys with cryptorchidism and normal boys.

**Methods:**

Total hCG and α-fetoprotein (AFP) values [12–16 weeks of gestation; from the double test for Down syndrome screening) were compared between cases of cryptorchidism and normal control boys who were matched for maternal age, maternal smoking, gestational age at time of hCG measurement (±1 day), birth weight and birth term. Measurements were performed in a single laboratory; values were expressed as absolute values (KU/L) and multiples of the median (MoM). Boys whose mothers had had a complicated pregnancy were excluded. Groups were compared using the Student’s *t* test. Log transformation was used to normalize hCG, MoM hCG, AFP and MoM AFP distribution, and values were expressed as geometric means (-1, + 1 tolerance factor).

**Results:**

Total hCG and MoM hCG levels were significantly lower in the 51 boys with cryptorchidism compared to 306 controls (21.4 (12.3; 37) KU/L vs 27.7 (15.9; 47.9) KU/L and 0.8 (0.5; 1.2) MoM vs 1.0 (0.6; 1.6) MoM, respectively, p < 0.01). By contrast, AFP and MoM AFP levels were similar between groups.

**Conclusion:**

This study showed a link between low maternal serum hCG levels and cryptorchidism in boys from uncomplicated pregnancy, while normal AFP levels indicated a normal fetoplacental unit. Whether these abnormalities were due to endogenous or exogenous factors remains to be determined.

## Background

Several studies have reported an unexplained increase in congenital malformations of the male reproductive tract, such as hypospadias and cryptorchidism [1]. Only a small proportion of hypospadias and cryptorchidism have been linked to genetic causes, while maternal pre-eclampsia, maternal diabetes and fetal size and premature birth were statistically associated with such malformations and therefore considered risk factors [2,3].

As male sexual differentiation is critically dependent on a balanced androgen to estrogen ratio, it has been postulated that male genital malformations may be related to in utero disturbances in exposure to estrogens and androgens [4]. One mechanism may be low fetal testosterone exposure, as testosterone and dihydrotestosterone are responsible for the closure of the urethal groove, the growth of the penis and migration of the testes [4-6]. Recent experimental studies in rats have identified a programming window for reproductive male masculinization preceding morphological differentiation [7]: interfering with androgen action during a window from E15.5 toE21.5 of rat gestation led to the highest rate of cryptorchidism (about 60%), as compared to interfering during an early windows only (E15.5-E17.5, 30% of cryptorchidism), or a late window only (E19.5-E21.5, no cryptorchidism). Based on the timings in rats, the programming window in humans would be 8–14 weeks of gestation [7]. In humans, testosterone is produced by the testes mainly in response to placental human chorionic gonadotrophin (hCG) during the first and second trimester of gestation , though regulation of placental hCG is complex and poorly understood [4,5]. Exposure to high estrogen concentrations could be another non exclusive mechanism [8]. Diethylstilbestrol (DES), an estrogenic endocrine disruptor chemical (EDC) has been shown to induce cryptorchidism in sons of mothers who took DES [8]. Although definitive proof of a physiological link between estrogen exposure and abnormal male differentiation is elusive, estrogens have been shown to inhibit placental gonadotropin releasing hormone (GnRH) [9], a key hormone stimulating hCG production [10]. Finally, all these studies suggest that abnormal placental hCG production could play a key role in disorders of sex differentation.

The present study used samples from Down syndrome screening, a double test combining total hCG and α-fetoprotein (AFP) measurements, which is offered to pregnant women in France during the second trimester of pregnancy. We showed that total hCG values were lower in cases of cryptorchidism in boys compared with those from normal control boys.

## Methods

### Patients

Prepubertal boys born between 1/1/99 and 31/12/07, at term (37 to 40 weeks of gestation) and presenting with uni or bilateral cryptorchidism (undescended testes which were not palpable in the scrotum or outside the external ring at 6 months of age or older) and referred to the Pediatric Surgery or Pediatric Endocrinology Units of Angers University Hospital were included in the study if their mothers had had blood collected at the University Hospital of Angers for Down syndrome screening. Boys who had also hypospadias or major anomalies of the genitalia were excluded from participating (since molecular causes could be expected with a higher likelihood in these cases). Boys whose mothers had had a complicated pregnancy were also excluded. The study was approved by the Institutional Review Board of Angers University Hospital. Affected boys were at least 6 months’ old at time of medical examination. Fifty one boys had cryptorchidism, including 3 bilateral cases, and 5 boys with associated micropenis (as defined by a stretched penile length < 3 cm between 1 and 12 months, and < 3.5 cm after 12 months). Anorchia was excluded (all had detectable antimullerian hormone concentration). In patients with bilateral cryptorchidism and/or associated micropenis, hypogonadotropic hypogonadism was excluded by testosterone measurement following an hCG test, as well as baseline inhibin B and antimullerian hormone measurements. The cases were compared with healthy boys born during the same time period in the Department of Obstetrics of Angers University Hospital, whose mothers had had blood collected at the University Hospital of Angers for double test screening (Down syndrome screening), and whose neonatal records reported no genital abnormalities: 306 controls babies were matched for maternal age (±0.5 yrs), maternal smoking (yes/no), gestational age at time of hCG measurement (±1 day), birth weight (±100 g), and week of gestation at birth ( ±1 wk) (i.e. 1 case per 6-8 controls).

### Laboratory analyses

All mothers had had blood drawn between 12 and 16 weeks of gestation, as determined from an early ultrasound scan. Total hCG and AFP were measured by immunofluorescence using a commercially available kit (Autodelfia Perkin Elmer, Wallac, Finalnd). Values were expressed as absolute values (kilounits/L; KU/L) and multiples of the median (MoM). hCG or AFP are expressed as MoM by dividing the hCG or AFP concentration by the median value for the appropriate week and day of gestation using the Multicalc software (Wallac, Finalnd).

### Statsitical analyses

Statistical analyses were performed using SPSS 16.0 software (SPSS Inc., Chicago, Illinois, USA). Log transformation was used to normalize hCG, MoM hCG, AFP and MoM AFP distribution, and values were expressed as geometric means (the antilogarithm of the mean of the logarithmically transformed data) (-1, + 1 tolerance factor) ( the antilogarithm of the standard deviation). Multiplying or dividing the geometric mean by the tolerance factor, or the square of the tolerance factor, is the equivalent of adding and subtracting one or two standard deviations in a normally distributed population. Other values were expressed as means (±SD) and groups were compared using the Student’s *t* test. Differences were considered to be significant if *P* < 0.05.

## Results

Characteristics of the 51 cases of cryptorchidism and their 306 matched controls are shown in Table 1. No difference in clinical as well as biological characteristics was seen between subjects with unilateral cryptorchidism, bilateral cryptorchidism, or cryptorchidism associated with micropenis (not shown).

**Table 1:** Characteristics of cases with cryptorchidism and their matched controls

	**Cryptorchidism**	**Matched controls**	**P**
Number of cases (N)	51	306	NS
Birth weight (kg)	3.31 ± 0.48	3.34 ± 0.46	NS
Birth term (weeks)	39.5 ± 1.2	39.5 ± 1.3	NS
Maternal age (years)	29.9 ± 4.4	29.5 ± 4.6	NS
Gestational age at time of screening (weeks)	13.6 ± 1.0	13.6 ± 1.0	NS
hCG (KU/L)^a^	21.4 (12.3; 37)	27.7 (15.9; 47.9)	< 0.01
MoM hCG^a^	0.8 (0.5; 1.2)	1.0 (0.6; 1.6)	< 0.01
AFP (KU/L)^a^	28.0 (18.6; 42.6)	28.8 (20.5; 40.5)	NS
MoM AFP^a^	0.9 (0.7; 1.4)	0.9 (0.7; 1.3)	NS

^a^geometric mean (-1, + 1 tolerance factor).

*hCG* human chorionic gonadotrophin, *AFP* α-fetoprotein, *MoM* multiples of the median.

Total hCG and MoM hCG levels were significantly lower in the 51 boys with cryptorchidism compared to 306 controls (21.4 (12.3; 37) KU/L vs 27.7 (15.9; 47.9) KU/L and 0.8 (0.5; 1.2) MoM vs 1.0 (0.6; 1.6) MoM, respectively, p < 0.01). By contrast, AFP and MoM AFP levels were similar between groups (Table 1).

## Discussion

Our data suggest a pathophysiologic link between placental hCG production and cryptorchidism. Relative hCG insufficiency could contribute to cryptorchidism, as hCG is known to stimulate fetal testicular androgen production with peak hCG levels at weeks 8-11 of gestation, after which hCG levels decline to 10–15% of peak concentrations from week 20 of gestation [4]. Of note, fetal circulating hCG is known to follow a similar pattern as maternal hCG, with circulating values approximately one third to one ninth of maternal values, fetal hCG being a more important ligand than luteinizing hormone (LH) for stimulating steroidogenesis up to 20 weeks of gestation [4]. The lower hCG values observed in mothers of boys with cryptorchidism could, therefore, lead to lower fetal testosterone production, thus contributing to the defect in testis migration. The association between placental hCG and minor genital anomalies has already been raised in a few studies [11-13]. No difference [11,13], or, at most, a trend toward a difference [12] was seen between affected boys and controls. However, these studies were associated with some limitations. Boys with cryptorchidism were mixed with subjects with hypospadias, the cohorts of cases and controls were small, serum samples were old stored samples, and the matching of subjects and controls was not precise, even for gestational age at time of hCG measurement (important considering that maternal hCG levels can change by up to 20% from one week to the next at this time of gestation) [14]. Finally, gestational age was not determined by early ultrasound scan in these studies, whereas ultrasound estimation of gestational age has been shown to significantly reduce the variance of the markers [15]. We therefore believe that a very precise matching for gestational age is likely to be critical, and this could explain the lack of power to differentiate hCG levels between the groups in these previous studies. In the present study, we excluded preterm birth as well as complicated pregnancy, and AFP levels were similar between cases and controls: we believe that the normal AFP levels indicated a normal fetoplacental unit, thus suggesting that the lower hCG values were not due to a dysfunctioning placenta. In one study, high AFP levels and low birth weight were both associated with cryptorchidism [16]. The authors suggested that this may reflect placental dysfunction, some aspect of which could contribute to cryptorchidism [16]. In this previous study, hCG was not measured : whether low hCG values due to a dysfunctioning placenta could have also contributed to cryptorchidism is therefore unknown.

Experimental evidences in rats have identified a programming window for reproductive male masculinization [7], and have shown that critical androgen action and physiological change in androgen levels did not need to occur at the same time. From the timing in rats, the corresponding programming window in humans would be 8–14 weeks of gestation whereas blood samples here were taken between gestational week 12 and 16, corresponding to the end of this window. Although it is likely that the altered hCG levels shown here could be present earlier in gestation, our study was not designed to ascertain this point nor directly measure androgen levels or action.

## Conclusion

In conclusion, this study showed a link between low maternal serum hCG levels and cryptorchidism in boys from uncomplicated pregnancy. Whether these abnomalities were due to endogenous (molecular origin or genetic susceptibility) or exogenous factors (exposure to environmental compounds) remains to be determined. Although speculative, the last hypothesis suggests that it could be crucial to evaluate further the effects of EDCs not only on the fetal testis, but also on the placenta.

## Competing interests

The authors declare that they have no competing interests.

## Authors’ contributions

CC participated in the acquisition of data and drafted the manuscript. HP, DW, and SR participated in the data collection and statistical analysis, and helped to draft the manuscript. RC conceived of the study, participated in the statistical analysis, and drafted the manuscript. All authors read and approved the final manuscript.

## Pre-publication history

The pre-publication history for this paper can be accessed here:

http://www.biomedcentral.com/1471-2431/14/191/prepub
